# Leveraging Bluetooth and GPS Sensors for Route-Level Passenger Origin–Destination Flow Estimation

**DOI:** 10.3390/s25082351

**Published:** 2025-04-08

**Authors:** Junming Xu, Zhenxing Pan, Cheng Zhang, Xiaoguang Yang

**Affiliations:** 1 Smart Urban Mobility Institute, University of Shanghai for Science and Technology, Shanghai 200093, China; 2235070526@st.usst.edu.cn (J.X.); yangxg@tongji.edu.cn (X.Y.); 2 Shanghai Seari Intelligent System Co., Ltd., Shanghai 200063, China; tj_pzx@126.com

**Keywords:** bus passenger, origin–destination flow estimation, Bluetooth sensing, multimodal data fusion, Fuzzy C-Means clustering

## Abstract

Accurate estimation of passenger origin–destination (OD) matrices is critical for optimizing public transportation systems, yet conventional methods face challenges, such as incomplete alighting data, high infrastructure costs, and privacy concerns. With existing GPS sensors and the additional deployment of a single low-cost Bluetooth sensor (10–20 US dollars) per bus, the proposed method can derive passenger OD flow without requiring passengers to tap in or tap out. The GPS sensor updates the bus locations, and the Bluetooth sensor receives signals from surrounding devices, including those onboard devices and nearby external devices. A Fuzzy C-Means clustering algorithm was employed to differentiate passenger and non-passenger devices based on detected indicators, such as detection frequency, signal strength, vehicular mobility, etc. Validation on Shanghai’s Fengpu BRT line demonstrated 91.22–96.02% accuracy in boarding proportion estimation and 95.18–95.52% for alighting during peak hours. Compared to the historical data-based method, the proposed method achieved higher similarity to ground truth and reduced the mean squared error by 12.89–69.95%.

## 1. Introduction

Accurate estimation of passenger origin–destination (OD) matrices is fundamental for optimizing public transportation systems, enabling evidence-based route planning, resource allocation, and service quality improvements [1]. Traditional methods face inherent limitations: manual surveys incur substantial labor costs and sampling biases [2], while automated fare collection (AFC) systems in open-loop payment environments frequently lack complete alighting information [3]. Although emerging technologies like Wi-Fi and video analytics have demonstrated potential to address these gaps, their implementation often encounters barriers, including high infrastructure costs [4] and privacy controversies [5]. These single-source approaches also exhibit inherent limitations—video analytics demands significant computational resources [3], while Wi-Fi-based solutions struggle with signal overdetection [6]—highlighting the need for multimodal integration.

This study proposes a novel, cost-effective methodology for OD estimation by integrating in-vehicle Bluetooth sensors and GPS data. Unlike existing approaches that rely on high-density sensor deployment or complex algorithms, our method requires only one low-cost Bluetooth sensor ($10–20 USD) per bus combined with widely available GPS and smart card datasets. Bluetooth technology offers advantages in device penetration (≈12–80% of passengers carry detectable devices) and spatial resolution [7,8], while GPS provides temporal–spatial vehicle context to filter non-passenger signals.

The proposed system simultaneously addresses two persistent challenges in transit OD analytics: (1) Significant reduction in hardware costs: This paper only requires one Bluetooth sensor (costing 10–20 yuan) plus existing GPS data. Compared with the scheme in [6] that requires the deployment of over 50 in-vehicle Wi-Fi probes (with a cost of 50 yuan per device), the cost per vehicle is reduced by 92–98%. When compared with the intelligent camera in the video surveillance scheme, which costs $5800 per vehicle as described in [9], the cost is reduced by 99.6%. (2) Estimation of OD flows between stations: By detecting the initial and final time of passengers’ addresses, the OD between stations for all passengers can be obtained. Previous studies only monitored the passenger flow of boarding and alighting at each station [10].

The remainder of this paper is structured as follows. Section 2 reviews the existing literature, discusses studies employing various methods for OD estimation, highlights the limitations of current approaches, and underscores the advantages of the present research. Section 3 proposes the feature calculation model and presents a clustering model designed to distinguish passengers from non-passengers. Subsequently, Section 4 develops the FCM algorithm for implementing the passenger/non-passenger differentiation clustering model. Section 5 conducts an empirical examination and provides a comparative analysis of the experimental results. Finally, Section 6 summarizes the key contributions and conclusions of this study.

## 2. Literature Review

### 2.1. Automated Fare Collection (AFC)-Based Approaches

Smart card data have become a primary OD estimation source due to their boarding time and location records. However, most AFC systems in bus networks only capture origins, requiring alighting stops to be inferred probabilistically. Lee et al. (2022) developed a trip-chaining algorithm using temporal patterns and historical records [11], achieving 60–74.9% destination matching accuracy. Similarly, Zhao et al. (2025) integrated AFC data with gravity models and entropy-weighted ensemble costs to estimate multimodal OD matrices, reducing mean absolute percentage errors (MAPE) to 12% [12]. While these methods improve upon manual surveys, their dependence on recurrent travel patterns limits applicability for irregular passengers [13].

### 2.2. Wireless Signal Detection Methods

Bluetooth and Wi-Fi sensing technologies enable passive OD estimation by tracking mobile devices. Early implementations by Kostakos et al. (2013) achieved 80% accuracy in reconstructing bus passenger flows using Bluetooth scanners, though device penetration rates (≈12%) constrained scalability [7]. Subsequent studies improved detection ranges and filtering algorithms: Pu et al. (2019) combined Fuzzy C-Means clustering with random forest regression to distinguish passengers from ambient signals, achieving 91% reidentification accuracy [10]. However, Wi-Fi/Bluetooth methods face challenges in dense urban environments due to signal interference and overdetection of non-passengers [6]. Recent case studies, further highlight the scalability challenges of dense signal-based approaches [14].

### 2.3. Video Analytics and IoT Solutions

Computer vision techniques extract OD data from surveillance footage by reidentifying passengers across stops. Zhang et al. (2025) [3] employed appearance feature matching and historical alighting probabilities, reducing root mean square errors (RMSE) to 0.5 persons/stop. While accurate, video-based systems require extensive computational resources and raise privacy concerns [15]. Complementary IoT approaches, such as NFC/BLE ticketing systems [16], provide precise OD data but necessitate passenger cooperation for check-in/check-out actions.

### 2.4. Hybrid and Emerging Methodologies

Recent studies integrate multiple data sources to overcome single-mode limitations. Owais (2024) combined deep learning with traffic sensor data to predict OD flows, achieving a 23% reduction in RMSE [9]. Similarly, Minea et al. (2020) fused Bluetooth triangulation with AI filtering for indoor passenger tracking, though hardware costs remained prohibitive for large-scale deployment [17]. Machine learning advancements, particularly self-supervised frameworks [18], show promise in handling incomplete datasets but require extensive training data [19].

### 2.5. Limitations of Existing Approaches

Current OD estimation methods exhibit three critical shortcomings:

- Data Sparsity: AFC-based models struggle with infrequent travelers, while wireless sensing suffers from device penetration variability [20].
- Infrastructure Costs: Video analytics and dense sensor networks (e.g., 50+ vehicle fleets in Demetrio et al., 2024 [6]) incur high deployment expenses [21,22].
- Real-Time Limitations: Most methods focus on historical analysis rather than dynamic OD monitoring [23].

**Figure 1:**
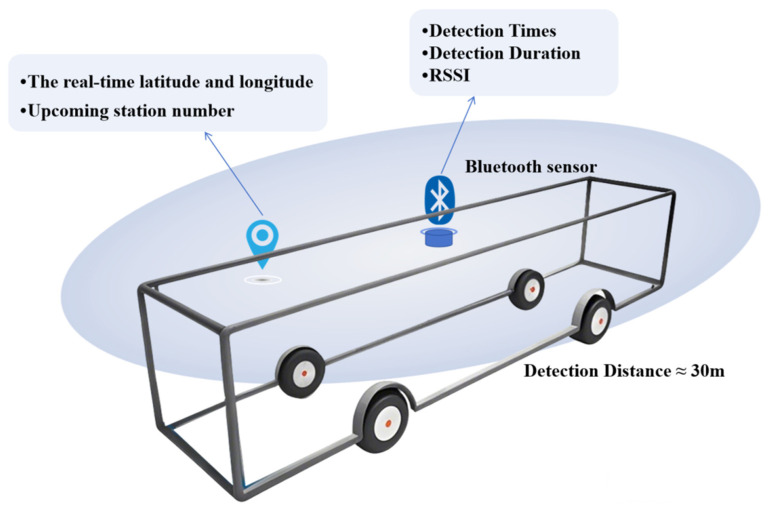
Illustration of the Bluetooth sensor.

The proposed Bluetooth–GPS fusion approach directly addresses these gaps by leveraging low-cost hardware and outputs the OD results between stations. By exploiting Bluetooth’s device ubiquity without requiring active passenger participation, this methodology enables scalable OD estimation—advancing beyond the constraints of prior single-modality systems. The Bluetooth sensor used in this article is shown in Figure 1.

## 3. Methodology

In this methodology section, the implementation of three sequential steps is described. Step 1 computes 9 features (4 MAC address features and 5 vehicular mobility features) to distinguish passengers from non-passengers. Step 2 employs the computed feature values as the input to a classification model, which is trained to identify passenger MAC addresses. Step 3 extracts boarding and alighting stations from the model-predicted passenger MAC addresses, thereby deriving the complete origin–destination (OD) matrices for all passengers. The methodology proposed in this study comprises three sequential phases:

Step 1: Feature Computation. Relevant information pertaining to Bluetooth devices detected during travel time intervals is acquired via in-vehicle Bluetooth detectors, while real-time geospatial coordinates (longitude and latitude) and subsequent station identifiers are obtained through onboard GPS detectors. The collected Bluetooth MAC address data and vehicle GPS data from the acquisition stage undergo preprocessing, after which a feature computation model is employed to derive eigenvalues for each Bluetooth MAC address. These eigenvalues serve as feature inputs for the clustering model designed to discriminate between passengers and non-passengers.

Step 2: Passenger–Non-passenger Differentiation. The computed eigenvalues of the Bluetooth MAC addresses are subjected to Fuzzy C-Means (FCM) clustering, yielding distinct clusters corresponding to passenger and non-passenger groups.

Step 3: Result Validation. For each Bluetooth MAC address within the passenger cluster, the initial and final detection timestamps are cross-referenced with the temporal markers in the vehicle GPS data. This enables the identification of the imminent station arrival time, which is subsequently designated as the boarding/alighting station for the corresponding address. By iteratively applying this procedure to all passenger MAC addresses, an estimated origin–destination (OD) matrix is generated. The accuracy of this estimation is rigorously evaluated against ground-truth OD information.

The parameters employed in this section are categorized in Table 1, organized according to the respective methodological components.

**Figure 2:**
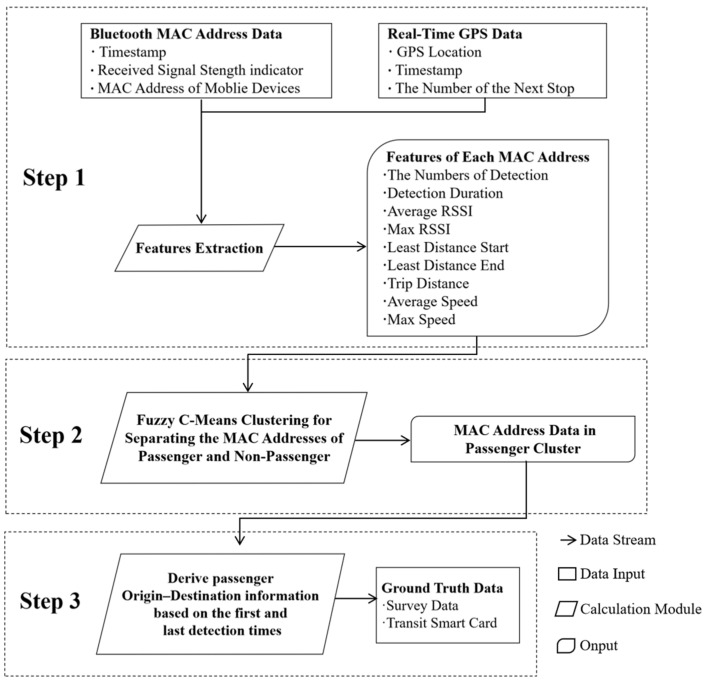
Methodological framework.

The schematic workflow of the proposed methodology is illustrated in the accompanying Figure 2.

### 3.1. Feature Computation

During the data acquisition phase, in-vehicle Bluetooth detectors are employed to identify Bluetooth-enabled devices (e.g., smartphones, laptops, Bluetooth earphones) within signal range during travel intervals. Concurrently, onboard GPS detectors capture real-time geospatial coordinates (longitude and latitude) and imminent station identifiers. The collected Bluetooth MAC address data and vehicle GPS data are processed through a feature computation model to derive characteristic metrics for each MAC address [24], as formalized below.

#### 3.1.1. MAC Address Features

1. Detection Times ($N_{m}$): Total number of detections for MAC address *m*, quantified as the count of data entries containing m in the Bluetooth dataset.
2. Detection Duration ($t_{m}$): Temporal span between the initial and final detection instances of MAC address *m*, measured in seconds (s). This metric is computed as follows:
  (1) $$tm=t_{last}^{m}-t_{first}^{m}$$
3. Mean RSSI ($\bar{R_{m}}$): The average received signal strength indication (RSSI) for MAC address *m*, measured in dBm. This metric characterizes the aggregate signal intensity level and is computed as follows:
  (2) $$\bar{R_{m}}=\frac{\sum_{t=t_{first}^{m}}^{t_{last}^{m}}R_{t}^{m}}{N_{m}}$$
4. Maximum RSSI ($R_{\max}^{m}$): The peak RSSI value observed for MAC address *m*, measured in dBm. This metric reflects either the closest physical proximity between the device and detector or the optimal signal interaction state, calculated as follows:
  (3) $$R_{\max}^{m}=\max\left\{R_{t_{first}^{m}}^{m},R_{t_{2}^{m}}^{m},\ldots ,R_{t_{last}^{m}}^{m}\right\}$$

#### 3.1.2. Vehicular Mobility Feature

To address temporal misalignment between Bluetooth and vehicular data sampling frequencies, temporal synchronization of Bluetooth MAC address timestamps with vehicular GPS timestamps is performed to geolocate MAC address observations. This ensures spatiotemporal consistency for subsequent feature computation. A temporal synchronization protocol is devised as follows:

(4) $$\forall t\in \tau_{BT}^{m},\tau_{match}^{m}\left(t\right)=\left\{\begin{array}{l} t\;\;\;\;\;\;\;\;\;\;if\;t\in \tau_{GPS}\\ \arg\min_{t\prime \in \tau_{GPS}}\left|t\prime -t\right|\;otherwise \end{array}\right.$$

Formula (4) specifies that, if a timestamp *t* in the detected timestamp set $\tau_{BT}^{m}$ of Bluetooth MAC address *m* exists within the vehicle GPS data’s timestamp set $\tau_{GPS}$, the corresponding real-time vehicle GPS data associated with timestamp *t* will be directly utilized. Otherwise, the algorithm selects timestamp *t*′ from vehicle timestamp set $\tau_{GPS}$ that exhibits the minimal absolute difference from *t*, thereby implementing a mapping operation using the temporally closest vehicle GPS data entry. This temporal alignment mechanism addresses the inconsistency in sampling frequencies between Bluetooth and vehicular GPS data acquisition systems, guaranteeing that each timestamp of every MAC address maintains a corresponding GPS coordinate mapping.

(5) $$T_{prev}=\left\{t\in \tau_{GPS}|t<t_{first}^{m},s_{next}\left(t_{first}^{m}\right)-1\right\}$$

(6) $$T_{next}=\left\{t\in \tau_{GPS}|t>t_{first}^{m},s_{next}\left(t_{first}^{m}\right)+1\right\}$$

(7) $$d_{front}^{m}=\left\{\begin{array}{l} \sum_{t=\max T_{prev}}^{t_{first}^{m}}\sqrt{{\left(lat_{t+1}-lat_{t}\right)}^{2}+{\left(lon_{t+1}-lon_{t}\right)}^{2}}\;if\;T_{prev}\neq \varphi \\ \infty \;\;\;\;\;\;\;\;\;\;\;\;\;\;\;otherwise \end{array}\right.$$

(8) $$d_{back}^{m}=\left\{\begin{array}{l} \sum_{t=t_{first}^{m}}^{\min T_{next}}\sqrt{{\left(lat_{t+1}-lat_{t}\right)}^{2}+{\left(lon_{t+1}-lon_{t}\right)}^{2}}\;if\;T_{next}\neq \varphi \\ \infty \;\;\;\;\;\;\;\;\;\;\;\;\;\;\;otherwise \end{array}\right.$$

(9) $$D_{start}^{m}=\min\left(d_{front}^{m},d_{back}^{m}\right)$$

1. Initial Detection Distance ($D_{start}^{m}$): The distance between the geographic location where Bluetooth MAC address *m* is initially detected and the nearest transportation hub, measured in meters (m). This parameter is calculated using the following formula:

The set *T_prev_* comprises all timestamps prior to $t_{first}^{m}$ that correspond to the vehicle’s position immediately preceding the target transportation node $s_{next}\left(t_{first}^{m}\right)$ − 1, while the set *T_next_* contains all timestamps subsequent to $t_{first}^{m}$ that align with the vehicle’s position immediately following the target node $s_{next}\left(t_{first}^{m}\right)$ + 1. For the computation of $d_{front}^{m}$, if *T_prev_* is non-empty, $d_{front}^{m}$ is calculated as the cumulative summation of geodesic distances between consecutive longitude and latitude coordinate points recorded from the last occurrence of $s_{next}\left(t_{first}^{m}\right)$ − 1 to $t_{first}^{m}$. Conversely, if *T_prev_* is empty, $d_{front}^{m}$ is assigned a value of infinity (∞). Similarly, for $d_{back}^{m}$, if *T_next_* is non-empty, $d_{back}^{m}$ is derived from the cumulative distance spanning from $t_{first}^{m}$ to the first occurrence of $s_{next}\left(t_{first}^{m}\right)$ + 1 in the trajectory data. If *T_next_* is empty, $d_{back}^{m}$ is likewise set to infinity (∞).

2. Final Detection Distance ($D_{end}^{m}$): The linear distance, measured in meters (m), between the geographic position of the last detected instance of MAC address *m* and its nearest transportation node. The computational methodology for this metric aligns with that of the initial detection distance ($D_{start}^{m}$).
3. Travel Distance ($S^{m}$): The total travel distance, in meters (m), traversed by the vehicle between the first and last detection timestamps of Bluetooth MAC address *m*. This parameter is derived through the following formula:
  (10) $$S^{m}=\sum_{t\in \left[t_{first}^{m},t_{end}^{m}\right]}\sqrt{{\left(lat_{t+1}-lat_{t}\right)}^{2}+{\left(lon_{t+1}-lon_{t}\right)}^{2}}$$

The cumulative geodesic distance is computed by iteratively summing the distances between consecutive longitude and latitude coordinate pairs recorded in the vehicle’s trajectory data across all timestamps spanning from $t_{first}^{m}$ to $t_{last}^{m}$.

4. Average Velocity ($\bar{v^{m}}$): The mean speed, expressed in meters per second (m·s^−1^), of the vehicle during the interval between the first $t_{first}^{m}$ and last $t_{last}^{m}$ detections of MAC address *m*. This parameter is derived through the following formula:
  (11) $$\bar{v^{m}}=\frac{S^{m}}{t_{last}^{m}-t_{first}^{m}}$$
5. Maximum Velocity ($v_{\max}^{m}$): The peak instantaneous speed, measured in meters per second (m·s^−1^), attained by the vehicle during the detection period of Bluetooth MAC address *m*. This parameter is derived through the following formula:
  (12) $$v_{\max}^{m}=\max\left\{\frac{\sqrt{{\left(lat_{t+1}-lat_{t}\right)}^{2}+{\left(lon_{t+1}-lon_{t}\right)}^{2}}}{\Delta t\left(t\right)}|t\in \left[t_{first}^{m},t_{last}^{m}\right]\right\}$$

For each time interval $\Delta t\left(t\right)$, the instantaneous velocity is calculated, and by traversing all the timestamps, the maximum value of the instantaneous velocity $v_{\max}^{m}$ is obtained.

### 3.2. Passenger–Non-Passenger Differentiation

#### 3.2.1. Objective Function

Let $X=\left\{x_{1},x_{2},\ldots ,x_{n}\right\}$ denote the dataset of n MAC addresses, where each $x_{n}\in R^{9}$ contains the 9 feature values. The FCM objective function $J_{\alpha }$ minimizes as follows:

(13) $$J_{\alpha }\left(U,V\right)=\sum_{i=1}^{c}\sum_{k=1}^{n}u_{ik}^{\alpha }{\left\|x_{k}-v_{i}\right\|}_{A}^{2}$$

where

c: Number of clusters (fixed at 2: passenger/non-passenger)

U = $\left[u_{\mathrm{ik}}\right]$: Fuzzy partition matrix ($u_{\mathrm{ik}}$ ∈ [0, 1])

V = $\left\{v_{1},v_{2}\right\}$: Cluster centroids

α: Fuzzification exponent (α > 1, typically 2)

$\|\;{\|}_{A}$: Mahalanobis distance with covariance matrix A

#### 3.2.2. Membership Update

Under probabilistic constraints $\sum_{i=1}^{c}u_{\mathrm{ik}}=1$, membership grades are computed as follows:

(14) $$u_{\mathrm{ik}}={\left[\sum_{j=1}^{c}{\left(\frac{{\left\|x_{k}-v_{i}\right\|}_{A}}{{\left\|x_{k}-v_{j}\right\|}_{A}}\right)}^{2/\left(\alpha -1\right)}\right]}^{-1}$$

Formula (14) indicates that, the smaller the Euclidean distance between $x_{k}$ and the center $v_{i}$ is, the larger the membership degree $u_{\mathrm{ik}}$ will be. The exponential term enhances the competition between classes, and α controls the fuzziness degree of the distribution of the membership degree.

#### 3.2.3. Centroid Update

Cluster centroids are recalculated as follows:

(15) $$v_{i}=\frac{\sum_{k=1}^{n}u_{ik}^{\alpha }x_{k}}{\sum_{k=1}^{n}u_{ik}^{\alpha }}$$

Centroids represent the weighted mean of all data points, where weights are the α-th power of memberships. This formulation emphasizes points with higher cluster affiliations.

## 4. Solution

### 4.1. Rationale for Clustering Algorithm Selection

The identification of passenger/non-passenger groups from MAC address signatures constitutes an unsupervised pattern recognition problem. Fuzzy C-Means (FCM) clustering is particularly appropriate for this task due to three inherent characteristics:

(a) Label ambiguity: Ground truth labels for passenger status are typically unavailable in transit monitoring systems. FCM autonomously discovers latent cluster structures without requiring pre-classified training data [25].
(b) Feature overlap: The nine-dimensional feature space exhibits nonlinear correlations between variables (e.g., detection duration vs. average speed). FCM’s probabilistic membership assignment handles overlapping cluster boundaries better than hard clustering methods [26].
(c) Dimensional complexity: With nine heterogeneous features spanning temporal, spatial, and signal strength domains, the Mahalanobis distance metric in FCM effectively weights feature contributions during cluster formation.

### 4.2. Pseudocode for Solving the Model

In this study, the algorithm is designed based on the assumption that the proportion of passengers carrying Bluetooth devices who get on and off at each station is evenly distributed overall. The parameters were determined based on previous research findings [10]. The fuzzification parameter α was set to 2, with the number of clusters c established as 2 to represent passenger and non-passenger groupings. The convergence threshold ε in the algorithm was configured at 10−5, while the maximum iteration count T was fixed at 1000 to ensure computational efficiency (Algorithm 1).
**Algorithm 1**: Passenger Classification via FCM ClusteringInput: Normalized feature matrix X, Cluster count c = 2, Fuzziness exponent α = 2.0, Convergence threshold ε = 10−5, Max iterations T = 1000Output: Membership matrix U, Cluster centroid matrix V1. Initialize $U^{\left(0\right)}$ with random memberships satisfying $\sum_{i=1}^{c}u_{\mathrm{ik}}=1$
2. Compute initial centroids $V^{\left(0\right)}$ via Equation (15)3. Repeat:a. Update $U^{\left(t+1\right)}$ using Equation (14)b. Update $V^{\left(t+1\right)}$ using Equation (15)c. Calculate $J_{\alpha }^{\left(t+1\right)}$ via Equation (13)d. Until $\left|J_{\alpha }^{\left(t+1\right)}-J_{m}^{\left(t\right)}\right|<\varepsilon$ or t>T
4. Assign labels: class($x_{k}$) = $\arg\max_{i}u_{\mathrm{ik}}$


## 5. Model Validation

To validate the effectiveness of the proposed methodology, a comprehensive case study was conducted through the deployment of Bluetooth detectors along authentic bus routes coupled with onboard GPS devices. This section begins with a description of the experimental background, followed by a systematic analysis of the empirical results.

### 5.1. Experimental Context

Bluetooth MAC address data and vehicle-mounted GPS datasets were collected along the operational Fengpu Bus Rapid Transit (BRT) corridor in Shanghai, China.

**Figure 3:**
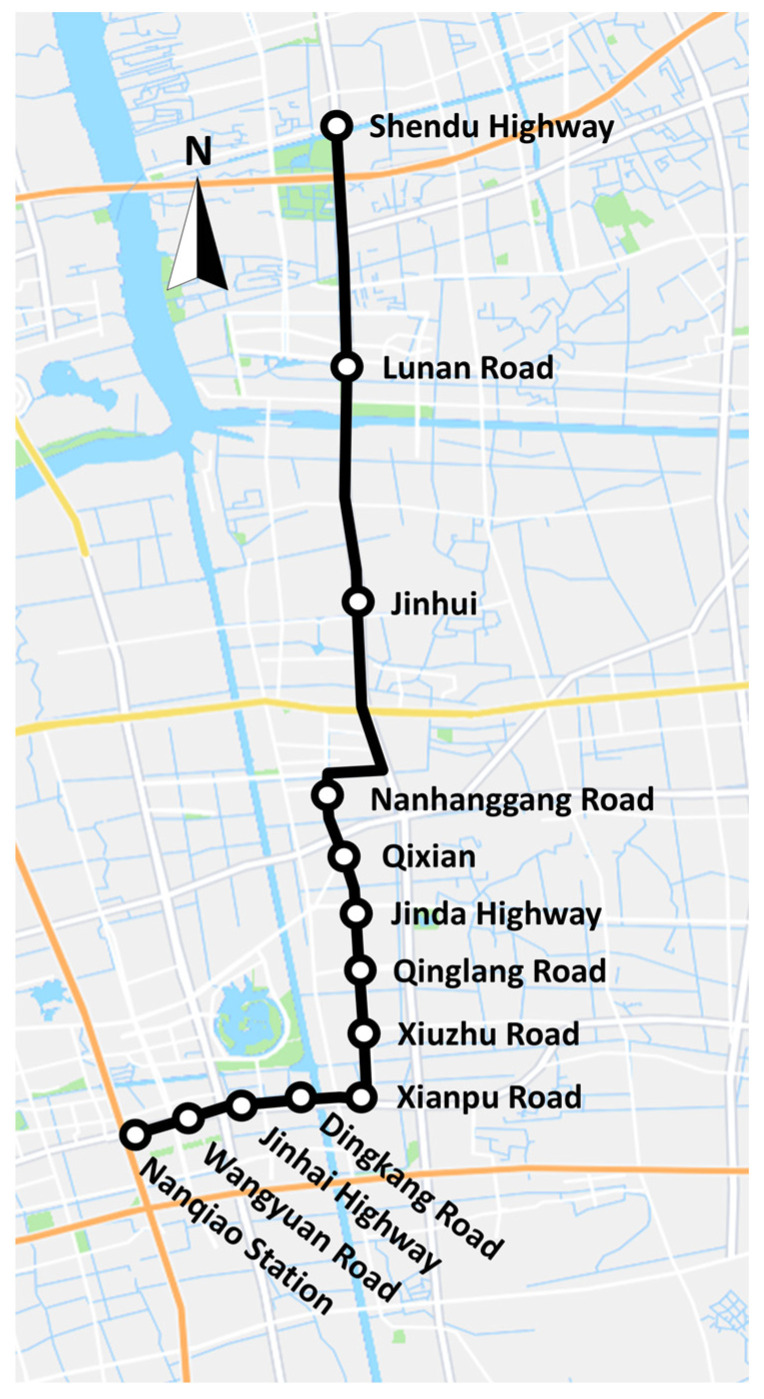
Fengpu BRT line.

As shown in Figure 3, the Fengpu BRT route comprises 13 stations. In January 2025, the proposed methodology underwent empirical validation through systematic evaluation in both directions of the BRT corridor. Two distinct operational scenarios were investigated: a morning peak-hour service (9:00–11:00) departing from Shendu Highway to Nanqiao Station, and an evening peak-hour service (17:00–19:00) operating along the same origin–destination corridor.

Implementation of the Proposed Method: The proposed method is automatically implemented through the following data inputs of the Fengpu Express Bus Line.

**Figure 4:**
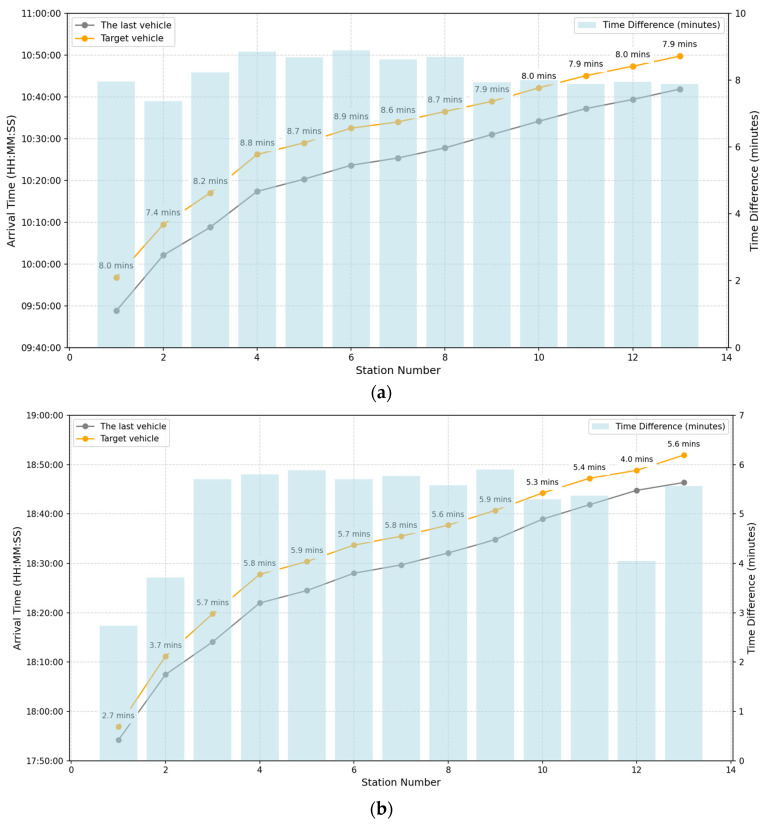
The arrival times of the target vehicle and the vehicle preceding it as well as the time difference between their arrivals. (**a**) shows the arrival times of the target vehicle and the preceding vehicle at each station during morning peak hours, while (**b**) displays the arrival times of the target vehicle and the preceding vehicle at each station during evening peak hours.

(1) Install Bluetooth detectors (located in the middle section of the vehicle) and collect Bluetooth MAC address data during the journey, which serve as the input for the proposed method. The collected Bluetooth MAC address data include information such as detection time, MAC address, Category (BT/BLE), Rssi, etc.
(2) Vehicle GPS data, which serve as the input for the proposed method. The collected vehicle GPS data include information such as detection time, longitude, latitude, direction of travel (indicating the driving direction of the vehicle), the station that the vehicle is about to arrive at, etc.
(3) Collection of ground truth: Firstly, determine the arrival times of the target vehicle for two trips and the vehicle preceding the target vehicle (i.e., the vehicle that arrives at each station earlier than the target vehicle) at each station, as shown in Figure 4a,b. Passengers whose arrival times at the stations are between those of the target vehicle and the vehicle preceding the target vehicle are regarded as passengers taking the target vehicle. For example, in Figure 4a, for Station 2, the arrival time of the vehicle preceding the target vehicle is 10:02:05, and the arrival time of the target vehicle is 10:09:27. The APC data of passengers who swipe their cards to enter the station at Station 2 within this time interval (including the time of entering and exiting the station and the station number of getting on and off the vehicle) are regarded as the data of passengers getting on the target vehicle at Station 2, and so on.

**Figure 5:**
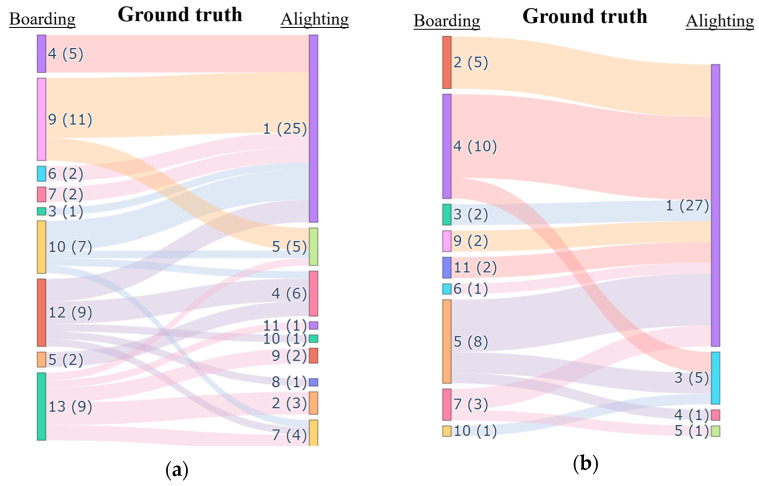
The ground truth of passenger flow OD. (**a**) shows the OD ground truth of the target vehicle during morning peak hours, while (**b**) displays the OD ground truth of the target vehicle during evening peak hours.

The ground truth origin–destination (OD) data for the two trips are obtained according to the above rules. To visualize the ground truth OD data, a Sankey diagram is adopted. As shown in the figure, on the left side are the alighting stations and the number of passengers alighting at each station, while on the right side are the boarding stations and the number of passengers boarding at each station. The lengths of the bar charts on both sides represent the passenger flow of boarding and alighting at the bus stops, and the numbers in parentheses indicate the number of boarding and alighting times. The width of the segments connecting the boarding and alighting stations represents the OD flow between the stations. Figure 5a,b respectively show two examples of bus trips during the morning and evening peak on 15 January 2024. One bus travels from Shendu Highway to Nanqiao Station during the morning peak (9:56–10:49), and the other bus travels in the same direction during the evening peak (17:56–18:48).

### 5.2. Experimental Results

To evaluate the accuracy of the proposed method in estimating the OD matrix, this section first compares the boarding and alighting ratios estimated by the proposed method with ground truth values, followed by performance comparisons with alternative OD estimation methods to demonstrate the effectiveness of our approach.

(1) To quantify the performance of the proposed method in predicting boarding and alighting ratios, a multi-stage computational approach is adopted. Specifically, the mean absolute error (MAE) between the BLE estimates and ground truth values is calculated, based on which the accuracy rate is derived. The detailed formulas and procedures are outlined as follows.

For comparative analysis of the BLE estimates and ground truth ratios, the true boarding ratio $P_{T}^{a}\left(i\right)$ and alighting ratio $P_{T}^{b}\left(i\right)$ at the *i*-th station are first computed separately, as expressed in Formula (16):

(16) $$P_{T}^{a}\left(i\right)=\frac{T_{i}^{a}}{\sum_{j=1}^{13}T_{j}^{a}},\;P_{T}^{b}\left(i\right)=\frac{T_{i}^{b}}{\sum_{j=1}^{13}T_{j}^{b}}$$

In Formula (16), $T_{i}^{a}$ and $T_{i}^{b}$ are the true values of the number of passengers boarding and alighting at the *i*-th station, respectively, and the denominator is the sum of the true values of the number of passengers boarding and alighting at the 13 stations.

Subsequently, calculate the proportion $P_{B}^{a}\left(i\right)$ of the number of passengers boarding detected by Bluetooth and the proportion $P_{B}^{b}\left(i\right)$ of the number of passengers alighting at the *i*-th station. The proportions of passengers boarding and alighting detected by Bluetooth are normalized as follows:

(17) $$P_{B}^{a}\left(i\right)=\frac{B_{i}^{a}}{\sum_{j=1}^{13}B_{j}^{a}},\;P_{B}^{b}\left(i\right)=\frac{B_{i}^{b}}{\sum_{j=1}^{13}B_{j}^{b}}$$

In Formula (17), $B_{i}^{a}$ and $B_{i}^{b}$ represent the number of passengers boarding and alighting detected by Bluetooth at the *i*-th station, respectively. The denominator is the total number of passengers boarding and alighting detected by Bluetooth at the 13 stations. The normalization process avoids the evaluation bias caused by the differences in traffic flow among stations, ensuring the comparability of the results.

To evaluate the consistency between the Bluetooth proportion and the true proportion, the mean absolute error (MAE) is adopted as the core indicator. The reason for choosing MAE instead of the mean squared error (MSE) is that MAE is less sensitive to extreme values and can more robustly reflect the average deviation of the proportion data [27].

(18) $$MAE_{a}=\frac{1}{13}\sum_{i=1}^{13}\left|P_{T}^{a}\left(i\right)-P_{B}^{a}\left(i\right)\right|$$

(19) $$MAE_{b}=\frac{1}{13}\sum_{i=1}^{13}\left|P_{T}^{b}\left(i\right)-P_{B}^{b}\left(i\right)\right|$$

In Formulas (18) and (19), *MAE_a_* is the mean absolute error of the proportion of the number of passengers boarding detected by Bluetooth, and *MAE_b_* is the mean absolute error of the proportion of the number of passengers alighting detected by Bluetooth.

Based on the MAE values of the Bluetooth detection proportions of passengers boarding and alighting, the corresponding accuracy rates are further calculated as follows:

(20) $$ACC_{a}=\left(1-MAE_{a}\right)\times 100\%$$

(21) $$ACC_{b}=\left(1-MAE_{b}\right)\times 100\%$$

In Formulas (20) and (21), *ACC_a_* represents the accuracy rate of the proportion of the number of passengers boarding detected by Bluetooth compared with the true value of the proportion of the number of passengers boarding, and *ACC_b_* represents the accuracy rate of the proportion of the number of passengers alighting detected by Bluetooth compared with the true value of the proportion of the number of passengers alighting.

The accuracy rates calculated by the above method are shown in Table 2.

During the morning peak, the accuracy rate of the proportion of the number of passengers boarding detected by this method is 91.22%, and the accuracy rate of the proportion of the number of passengers alighting is 96.02%. During the evening peak, the accuracy rate of the proportion of the number of passengers boarding detected by this method is 95.18%, and the accuracy rate of the proportion of the number of passengers alighting is 95.52%. The results indicate that this method has a high degree of consistency with the true values in predicting the proportions of the number of passengers boarding and alighting, especially in the detection of the proportion of the number of passengers alighting.

(2) Comparison with the method [28] of estimating the origin–destination (OD) based on historical boarding and alighting data (HD-based): In this part, we will select three indicators that are widely used to evaluate the prediction accuracy, namely the mean squared error (MSE), the mean absolute error (MAE), and the similarity, to analyze the OD values estimated by different methods. Moreover, during the calculation process, the data of Station 1 and Station 13 are excluded to avoid the interference of possible special situations on the results.

(a) Mean squared error (MSE). The mean squared error is a commonly used indicator for measuring the degree of deviation between the predicted values and the true values. By averaging the squares of the prediction errors, it can comprehensively reflect the overall deviation of the prediction results. Its calculation formula is shown as follows:

(22) $$MSE=\frac{1}{n}\sum_{i=1}^{n}{\left(y_{i}-{\hat{y}}_{i}\right)}^{2}$$

In the Formula (22), *n* represents the sample size, $y_{i}$ represents the true value of the *i*-th sample, and ${\hat{y}}_{i}$ is the estimated value of the *i*-th sample.

(b) Mean absolute error (MAE). The mean absolute error is another important indicator for evaluating the prediction accuracy. It directly calculates the average of the absolute errors between the predicted values and the true values and intuitively reflects the average degree of deviation between the prediction results and the true values. Its calculation formula is shown as follows:

(23) $$MAE=\frac{1}{n}\sum_{i=1}^{n}\left|y_{i}-{\hat{y}}_{i}\right|$$

(c) Similarity. The cosine similarity is employed to measure the degree of similarity between the estimated origin–destination (OD) matrices. The reason for using the cosine similarity is that it can effectively quantify the angular similarity between two vectors without being affected by the magnitudes of the vectors. This is particularly useful when comparing the OD flow patterns estimated by different methods. In the context of OD matrix estimation, vectors represent the passenger flow distribution between different origin–destination pairs. The cosine similarity can highlight the similarity of flow patterns without being overly influenced by the absolute values of the flow volume, thus more meaningfully measuring the similarity of the estimated OD structures. The calculation formula for the cosine similarity is as follows:

(24) $$\cos\left(\theta \right)=\frac{\vec{A}\cdot \vec{B}}{\left\|\vec{A}\right\|\cdot \left\|\vec{B}\right\|}=\frac{\sum_{i=1}^{n}A_{i}B_{i}}{\sqrt{\sum_{i=1}^{n}A_{i}^{2}}\sqrt{\sum_{i=1}^{n}B_{i}^{2}}}$$

In Formula (24), $\vec{A}$ and $\vec{B}$ are vectors representing the OD matrices estimated by different methods (e.g., Bluetooth-based (BT-based) estimation and historical boarding-alighting data-based (HD-based) estimation). $A_{i}$ and $B_{i}$ are the elements at the *i*-th position in vectors $\vec{A}$ and $\vec{B}$, respectively, and *n* is the number of elements in the vectors, which is the number of origin–destination pairs.

The numerator $\sum_{i=1}^{n}A_{i}B_{i}$ calculates the dot product of the two vectors, measuring the extent to which the values at each corresponding position of the two vectors point in the same direction. The larger the dot product, the more similar the values at the corresponding positions are. The denominator $\sqrt{\sum_{i=1}^{n}A_{i}^{2}}\sqrt{\sum_{i=1}^{n}B_{i}^{2}}$ normalizes the vectors to ensure that the value of the cosine similarity lies within the range of [−1, 1]. When the two vectors are identical, the cosine similarity is 1, indicating a perfect match; when they are orthogonal (completely different in direction), the cosine similarity is 0; when they are in exactly opposite directions, the cosine similarity is −1. 

**Figure 6:**
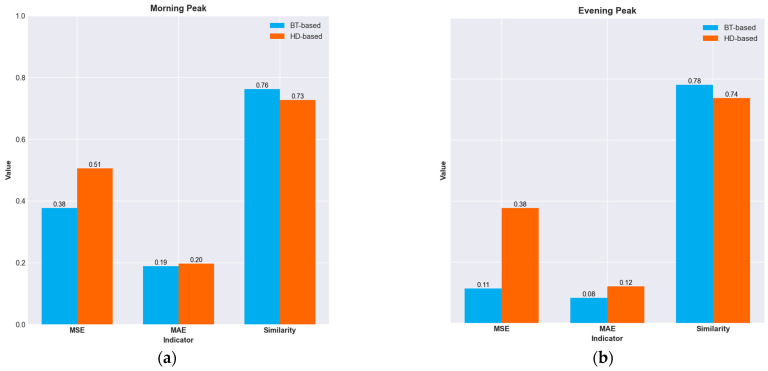
The performance of passenger flow origin–destination (OD) estimation methods in different scenarios. (**a**) shows the performance comparison of different OD estimation methods during morning peak hours, while (**b**) displays the performance comparison of different OD estimation methods during evening peak hours.

Figure 6a,b demonstrate the performance of different OD estimation methods across varying operational scenarios.

As shown in Table 3, in the context of the morning peak period, the MSE index of the BT-based method proposed in this paper is 0.377, which is 0.129 lower than that of the HD-based method (0.506), thus demonstrating superior error control capability. Regarding the MAE index, although the difference between the two methods has narrowed (0.189 vs. 0.197), the BT-based method still maintains a relative advantage. It is noteworthy that the difference in the similarity index is the most pronounced. The BT-based method achieves a similarity of 0.763, significantly outperforming the HD-based method’s 0.727. This indicates that, when dealing with the complex traffic flow patterns during the morning peak, the BT-based method exhibits stronger anti-interference ability and adaptability.

Upon further analysis of the performance differences across different time periods, it is found that the BT-based method demonstrates more outstanding estimation performance during the evening peak period. Its MSE index is merely 0.114, which is 69.95% lower than that of the HD-based method (0.378). This significant difference validates that the BT algorithm has better parameter convergence during the evening peak period when the commuting regularity is higher. Meanwhile, in terms of the MAE and similarity indices, the BT-based method achieves excellent results of 0.083 and 0.782, respectively, which are 31.9% lower in MAE and 5.96% higher in similarity than those of the HD-based method (0.121 MAE and 0.738 similarity), further confirming its robustness in traffic state estimation.

It is particularly important to point out that the HD-based method shows significant fluctuations in the similarity index, reaching 0.727 and 0.738 during the morning and evening peaks, respectively, which reflects that this method is highly sensitive to scenarios with sudden changes in traffic flow. In contrast, the similarity performance of the BT-based method during the two peak periods ranges between 0.763 and 0.782, showing strong stability.

## 6. Conclusions

This paper proposes a method for estimating the origin–destination (OD) matrix of bus passengers based on the fusion of multi-source data from Bluetooth and GPS. Through the deployment of low-cost hardware and the design of intelligent algorithms, it effectively addresses the limitations of traditional OD estimation methods in terms of data integrity and real-time performance. The experimental results show that this method demonstrates significant performance advantages in real bus operation scenarios, providing a new technological path for the intelligent management of urban public transportation. Theoretically, this paper constructs a multi-dimensional feature calculation model that incorporates MAC address features and vehicle driving features. Through the Fuzzy C-Means (FCM) clustering algorithm, it achieves accurate differentiation between passenger and non-passenger devices. Compared with traditional inference methods based on historical travel patterns, this method achieves an accuracy rate of 0.9122–0.9602 for estimating the proportion of boarding passengers and 0.9518–0.9552 for estimating the proportion of alighting passengers during the morning and evening rush hours, verifying the adaptability of multi-source data fusion to complex traffic scenarios.

In terms of practical applications, this method adopts a lightweight deployment scheme of installing one low-cost Bluetooth sensor per vehicle (cost 10–20 US dollars), which significantly reduces the high investment required by traditional video analysis or dense sensor networks. Empirical analysis shows that, compared with the historical data (HD) method based on historical data, this method achieves a similarityof 0.763–0.782 during peak hours, outperforming the HD-based method’s 0.727–0.738, providing a feasible technical option for bus companies to implement real-time passenger flow monitoring and dynamic dispatching optimization. In addition, the system’s ability to update the OD data at a minute-level can effectively support intelligent transportation application scenarios, such as early warning for sudden large passenger flows and signal control for bus priority.

Despite the aforementioned achievements, this study still has certain limitations. Firstly, the dependence of feature calculation on vehicle driving parameters may affect performance in areas with low GPS positioning accuracy. Secondly, spatiotemporal fluctuations in Bluetooth device penetration rates may introduce sampling bias for specific demographic groups. Future research will focus on four strategic optimizations: (1) Developing adaptive clustering algorithms with sliding time windows and online learning mechanisms to address dynamic device penetration rates; (2) Constructing multimodal data fusion frameworks that integrate behavioral features from onboard cameras with spatial trajectory characteristics from Bluetooth signals for comprehensive passenger profiling; (3) Enhancing methodological generalizability through multi-route validation (categorized by spatial attributes, including downtown areas, suburbs, transportation hubs, and large facilities like hospitals/schools) and multi-temporal experiments (covering peak hours, off-peak periods, and holidays) [29]; (4) Conducting quantitative analysis of external factors, including device variability (sensor drift, firmware heterogeneity) and signal interference (multipath effects, electromagnetic noise), with error propagation modeling to improve data reliability. Through continuous algorithmic refinement, this method is expected to become a core technical component supporting the digital transformation of urban bus systems. 

## Figures and Tables

**Table 1:** Notations.

Feature Computation	
*m*	Bluetooth MAC address
*t*	Timestamp
$Lon_{t}^{m}$	Longitude of Bluetooth MAC address *m* detected at timestamp *t*
$Lat_{t}^{m}$	Latitude of Bluetooth MAC address *m* detected at timestamp *t*
$N_{m}$	Total detection count of Bluetooth MAC address *m*
$t_{m}$	Detection duration of Bluetooth MAC address *m*
$t_{last}^{m}$	Last detection timestamp *t* for Bluetooth MAC address *m*
$t_{first}^{m}$	First detection timestamp *t* for Bluetooth MAC address *m*
$\bar{R^{m}}$	Average RSSI value of Bluetooth MAC address *m*
$R_{\max}^{m}$	Maximum RSSI value of Bluetooth MAC address *m*
$R_{t}^{m}$	RSSI value recorded when detecting Bluetooth MAC address *m* at timestamp *t*
$\tau_{GPS}$	Timestamp set of vehicle GPS data
$\tau_{BT}^{m}$	Bluetooth timestamp set of MAC address *m*
$\tau_{match}^{m}$	Matched timestamp set after aligning Bluetooth timestamps with vehicle GPS timestamps
$s_{next}\left(t\right)$	Upcoming station number of the vehicle at timestamp *t*
$T_{prev}$	Timestamp set corresponding to (upcoming station number−1) before first detection time
$T_{next}$	Timestamp set corresponding to (upcoming station number + 1) before first detection time
$d_{front}^{m}$	Distance from preceding station for Bluetooth MAC address *m* at timestamp *t*
$d_{back}^{m}$	Distance to subsequent station for Bluetooth MAC address *m* at timestamp *t*
$D_{first}^{m}$	Minimum distance to the nearest station during first detection of Bluetooth MAC address *m*
$D_{end}^{m}$	Minimum distance to the nearest station during last detection of Bluetooth MAC address *m*
$S^{m}$	Total travel distance between first and last detection instances of Bluetooth MAC address *m*
$\bar{v^{m}}$	Average travel speed during detection period of Bluetooth MAC address *m*
$v_{\max}^{m}$	Maximum instantaneous speed during detection period of Bluetooth MAC address *m*
$\Delta t\left(t\right)$	Time interval between consecutive timestamps *t* + 1 and *t*
**Passenger–Non-passenger Differentiation**	
$J_{\alpha }$	Objective function
*U*	Membership matrix
*V*	Cluster centroid matrix
$x_{k}$	k-th data sample
$u_{ik}$	Membership degree of sample k to cluster i
$v_{i}$	i-th cluster centroid
*n*	Number of MAC addresses
α	Fuzzification exponent
*c*	Number of clusters (fixed at 2: passenger/non-passenger)
*A*	Covariance matrix
*X*	Input feature matrix
**Result Validation**	
*i*	The *i*-th station
$T_{i}^{a}$	Ground-truth boarding count at station *i*
$T_{i}^{b}$	Ground-truth alighting count at station *i*
$B_{i}^{a}$	Bluetooth-detected boarding count at station *i*
$B_{i}^{b}$	Bluetooth-detected alighting count at station *i*
$P_{T}^{a}\left(i\right)$	Ground-truth boarding proportion at station *i*
$P_{T}^{b}\left(i\right)$	Ground-truth alighting proportion at station *i*
$P_{B}^{a}\left(i\right)$	Bluetooth-detected boarding proportion at station *i*
$P_{B}^{b}\left(i\right)$	Bluetooth-detected alighting proportion at station *i*

**Table 2:** The accuracy rate of the proportion of the number of alighting passengers and boarding passengers detected by Bluetooth.

Scenario	*ACC_a_*	*ACC_b_*
Morning Peak	91.22%	96.02%
Evening Peak	95.18%	95.52%

**Table 3:** The performance of passenger flow origin–destination (OD) estimation methods in different scenarios.

Scenario	OD Estimation Method	MSE	MAE	Similarity
Morning Peak	BT-based	0.377	0.189	0.763
	HD-based	0.506	0.197	0.727
Evening Peak	BT-based	0.114	0.083	0.782
	HD-based	0.378	0.121	0.738

## Data Availability

The raw data supporting the conclusions of this article will be made available by the authors on request.

## Abbreviations

The following abbreviations are used in this manuscript:

BT	Bluetooth
FCM	Fuzzy C-Means
MAE	Mean Absolute Error
MSE	Mean Squared Error
HD	Historical boarding and alighting data
